# Syphilis of Stomach

**Published:** 1916-10

**Authors:** 


**Syphilis of Stomach.** Georges Hayem, Bull. de l'Acad. de Med., Feb. 29, reports a case and mentions a previous one, in which the gastric analysis and tumor, which on palpation seemed continuous with the hypertrophied liver, suggested cancer. The pain was more marked than in a properly treated gastric cancer case. The gastric lesion developed 40 years after the primary infection and was relieved by mercurial treatment.

